# The Thaw Case

**Published:** 1907-05

**Authors:** 


**A Monthly Review of Medicine and Surgery.**

*EDITOR:*

WILLIAM WARREN POTTER, M. D.

All communications, whether of a literary or business nature, books for review and exchanges, should be addressed to the editor 238 DELAWARE AVE., BUFFALO, N. Y.

VOL. LXII. MAY, 1907. NO. 10

### The Thaw Case.

NOW that the unsavory Thaw case has been temporarily shelved by the disagreement of the jury which listened for seven weeks to the salacious details of a chorus girl's frivolities, to place the description in the category of mildly stated conditions; now that they have waded through the confusing opinions of men of science whose mental studies of the mind of the prisoner were kaleidoscopic, and have had inflicted upon them a two day's summing up on the part of a gentleman of perfervid eloquence and maudlin mushiness, there are several points accentuated by the trial which demand consideration—and radical change. Without going into the intimate details of the testimony to any great degree, one must be impressed with the fact that two great evils are existent in present day methods of criminal trial. One, and the foremost, is the absolute ill-treatment accorded jurymen. Here, after seven weeks of intolerant bickerings and squabbles, in the bitterness of which the prisoner and the central idea of criminal trial were lost sight of, the jury was locked up for forty-eight hours. True, they were fed, but they were given no opportunity for sleep; they did not have a change of clothing and from weariness they were quickened to irritability and thence to the verge of violence, and the disgraceful spectacle was presented of two men with hands raised against one another over the question—not as to the guilt or innocence of the prisoner, but as to whether the views expressed and the ballot cast by one of the jurors was honest. It was not a serious difference based on serious argument; it was merely an explosion of irritability which culminated after many hours of torturing confinement. No man, except he be an unusual almost uncanny creation, can be expected to act with judgment and wisdom unless he have physical rest, and this fact was never better illustrated than in the trial which has just been finished at great expense and without satisfactory result.

The next most prominent feature demanding change, and one which will very likely be corrected by a bill now before the legislature, is the law governing the giving of expert testimony. Here was a picture of a prosecution and a defense, both financially able and willing to take fullest advantage of the rules governing expert evidence and there was presented the sorry spectacle of sober, serious, scientific, savants sitting suavely in court awaiting their turn to go on the stand and give evidence for their hire. One cannot blame the layman for asking why, if a man is sane or insane the fact cannot be coldly stated by experts; and again the query is raised, of what use is a commission in lunacy which is composed of two laymen and one physician, especially when it would appear from the published reports that the latter's opinion was either forced or ignored altogether? On the Thaw side were several eminent men whose years have been spent in the study of insanity; on the side of the prosecution were men equally learned. Yet, Thaw's experts testified to sanity at the present time and swore that when Stanford White was killed the prisoner did not know the nature of his act. Fine distinctions from hearsay evidence!

District Attorney Jerome presented experts who testified diametrically opposite; and it must be said after a careful review of the evidence that the latter gentlemen were in better position than their learned opponents. The entire case from the Thaw side savored, from the very beginning, of a too fond reliance on the power of money and its ability to purchase what was needed, even brains. The declaration of the suddenly rich financier who decried college education for his sons, saying they didn't need brains, he had money enough to buy all they needed, appears to have been engirdled with truth by this trial's incidents. The logical outcome of this would appear to be the appointment by the court of a commission of experts, medical and legal, whose opinions would be at the service of each side; a commission which should be impartially at the disposal of the court, and made up of men of unquestioned integrity. The expense should be borne by the state and the pay should be sufficient.

Viewed dispassionately, the attitude of District Attorney Jerome must commend itself to all who are not slushily inclined to wallow in incubated sentiment over the alleged wrongs of a poor chorus girl and the mock heroic defense of a selfish, self-centered and perverted mind. The real issue which the district atttorney sought always to hold before the jury was that a murder had been committed; that the man who did the killing was guilty of murder and should be punished. If sane, then let him follow the route taken by other murderers who have been convicted. If insane, then the proper place for him was an asylum for the criminal insane. That was Jerome's attitude and it cannot but commend itself to all serious minded people whether they be lay or professional men. Jerome's honesty of purpose, in spite of the fact that it has been called a trick, by those who bow before the shrine of the unwritten law, and many of them do that just because it looks well, and makes a noise in argument, is best shown by his demand for a commission in lunacy. Jerome believes that Thaw is of that type which is dangerous to society. There is much in the evidence to prove this to be so and the fact that eight jurors voted for conviction of murder in the first degree is significant, as are the facts that not one juror paid much attention to the expert testimony or believed the brilliant and pathetic tale of sexual assault recited by Evelyn Nesbit Thaw on the witness stand; the tale of her drugging and her vividly described rape. Surely, no one who has paid any serious attention to the study of sex and crime could think otherwise than that she had been carefully schooled in the recital of that woesome story; so full of probability and realism and yet so sadly lacking in the essentials which would have made it believable, the essentials which are at once sought by the student of sex.

Thaw will be tried again and the case will be tried on its merits as a case of murder without brain storms or other confusing elements, it is hoped. Stanford White was shot. Harry Thaw shot him. Murder is excusable in self-defense in its broadest sense. White was shot in the back. The excuse was that at a time when his murderer did not even know the girl, he had raped by force; the girl whom Thaw afterward made his wife after dragging her through the slums of Europe as his mistress. The elements of self-defense are lacking; the home-defense is lacking, and apparently the case assumes the qualities which Jerome ascribed to it in his summing up—a vulgar Tenderloin murder.

To which might be added that its basis is sexual perversion.

